# Subgroup detection-based dental caries status and inequalities trend exploration: A nationwide, 10-year-repeated cross-sectional study

**DOI:** 10.3389/fpubh.2022.916878

**Published:** 2022-08-12

**Authors:** Jie He, Hongyuan Liang, Jian Kang, Chao Yuan

**Affiliations:** ^1^Department of Biostatistics, School of Public Health, Peking University Health Science Center, Beijing, China; ^2^Department of Medical Administration, Peking University School and Hospital of Stomatology, National Center of Stomatology, National Clinical Research Center for Oral Diseases, National Engineering Research Center of Oral Biomaterials and Digital Medical Devices, Beijing, China; ^3^Department of Biostatistics, University of Michigan, Ann Arbor, MI, United States; ^4^Department of Preventive Dentistry, Peking University School and Hospital of Stomatology, National Center of Stomatology, National Clinical Research Center for Oral Diseases, National Engineering Research Center of Oral Biomaterials and Digital Medical Devices, Beijing, China

**Keywords:** repeated cross-sectional studies, public health dentistry, epidemiology, inequalities, Poisson mixture regression

## Abstract

**Background:**

The goal of this study was to identify potentially important factors for the dental health though heterogeneous effects of risk factors within Chinese adolescent populations with different characteristics by analyzing the repeated cross-sectional data collected in the 3rd (2005) and 4th (2015) National Oral Health Survey.

**Methods:**

We studied the relationships between the decayed, missing and filled permanent teeth (DMFT) score, which was a discrete value, with the caries risk factors (region, census type, gender, only child or not, parents' education level, tooth bushing, dentist visit history, knowledge score, sugar intake, and pit-and-fissure sealants status), though the Poisson mixture regression model, which could identify subgroups among the full population and estimate the heterogeneous effects of risk factors simultaneously. We performed a series of tests and trend analysis based on the model fitting results to explore the primary causes for the dental caries issue clearly and intuitively.

**Results:**

A total of 39,049 teenagers aged 12 years were involved in the analysis. The Poisson mixture regression model clustered all individuals into three subgroups, where the mean values (standard deviations) of DMFT were 0.18 (0.56), 1.31 (1.49), and 2.91 (1.89), respectively. Model fitting results indicated that the heterogeneous effects of the involved risk factors were significant. In addition, we also found significant differences in the distributions and trends of DMFT within different categories of selected risk factors (region, census type, gender and dentist visiting history) from the projection analysis results. The estimated and projected proportions showed that the proportion of high caries risk population in the southwestern region increased by 31.8%, and will become even more severe as it will be the major component of high caries risk population in 2025.

**Conclusions:**

We found that the trends for the developments and changes of dental caries within populations with different characteristics were inequality. The regional difference is the primary factor for diversified changes in DMFT. The findings in this study provide support for intervention and prevention policies for the deterioration of dental caries risk within different adolescent populations.

## Introduction

Currently, dental caries has become the most prevalent disease, and its incidence is the second-highest in the Global Burden of Disease (1). The prevalence and experience of dental caries have declined for decades in many developed countries (2). However, existing evidence indicates that the global rate of dental caries is still increasing, and the situation of polarization is becoming increasingly severe (3). Taking China as an example, the proportions of permanent dental caries for 12-year-old children in 2005 and 2015 were 28.9 and 38.5%, respectively (4, 5). It is obvious that the status of dental caries has worsened during the past decade (6). One major reason for the prevalence of permanent and deciduous caries is sociodemographic imbalance (7). For instance, a relevant study on dental caries shows that the DMFT status for Chinese teenagers differs significantly among different regions (8). Moreover, the oral behaviors of adolescents, including toothbrushing habits, sugary food consumption and dental attendance, also vary among different provinces as well as urban and rural areas (9, 10). According to previous studies in China, the prevalence of dental caries is greatly influenced by regional factors (11, 12). Adolescents in the western region of China still had the highest prevalence of dental caries. The unequal distribution of economic development levels and human resources for oral health is be a major reason for the regional differences in dental caries (13). In addition, other studies show that the dental caries status of 12-year-old adolescents depends on socioeconomic factors (14, 15), education in parents (16), and oral hygiene behaviors (17–19).

The influence of risk factors on dental caries exhibits heterogeneity (12). Specifically, among populations with different individualized characteristics and demographic statuses, the characteristics and induction factors for dental caries status are usually different. Thus, identifying the heterogeneous effects is essential for us to find primary reasons for the prevalence of dental caries.

The repeated national cross-sectional oral health survey has been conducted regular in China for every 10 years since 2005. However, existing studies on cross-sectional dental caries data could not effectively identify the effects of risk factors on dental caries status between the repeated cross-sectional survey, let alone incorporate the heterogeneity issue. Thus, we aimed to discover the heterogeneous relationships between the dental caries and the risk factors for interest based on 10-year repeated cross-sectional data. On the one hand, the analysis results can help us obtain trends for the developments and changes of dental caries within populations with different characteristics. On the other hand, combining all analysis results in different populations, we could propose some comprehensive suggestions on reformation and policy innovation for dental public health, which is essential for improving the current dental caries situation.

## Materials and methods

### Description of data

This repeated cross-sectional study used the data collected from the 3rd (2005) and 4th (2015) National Oral Health Survey in China. All 31 provinces in mainland China participated in this survey, except for Tibet. In this study, we only used the observations with both clinical examination and questionnaire records in the 12-year-old teenager group (11,228 in 2005 and 27,821 in 2015) in the data analysis procedure.

Post-stratification weights were used to adjust for the differences in the age-by-sex-by-location-by-province distributions between the sample and the general populations in 31 provinces involved in the study, which is consistent with the 5th National Demographic Census in 2000 (20) and the 6th National Demographic Census in 2010 (12, 21).

The number of DMFT is an important index for evaluating the overall caries status and experience. We chose the factors (region, census type, gender, only child or not, parents' education level, tooth bushing, dentist visit history, knowledge score, sugar intake, and pit-and-fissure sealant status) as potentially important factors for dental caries and performed further statistical analysis through the Poisson mixture regression model.

### Patient and public involvement

All participants in this study were selected using a multistage stratified cluster sampling method, and written informed consent for participation in this study was provided. Ethical clearances of both surveys in 2005 and 2015 were approved by the Stomatological Ethics Committee of the Chinese Stomatological Association.

### Model and estimation

The Poisson mixture regression model is an efficient method for detecting the heterogeneity effects, which classifies all individuals into subgroups automatically and makes statistical inferences on the heterogeneous effects of all risk factors within each subgroup at the same time. It is flexible in modeling the inflated zero count measure for dental decay and capturing the subgroup-specified influence of risk factors on dental caries.

The whole statistical inference procedure was completed by the R package “FlexMix” (22–24). Except for all risk factors, to obtain the developing trend of heterogeneous effects, we also incorporated the time indicator (0-2005, 1-2015) as well as its interaction terms with all 10 risk factors in the Poisson mixture regression model. An optimized group number was selected from a series of prespecified group numbers through the Bayesian information criterion (BIC) (25, 26), and the whole computation procedure can be finished by the modified EM algorithm. The specific formula and other detailed information about the Poisson mixture regression model is available in the Appendix. Additional explanation for all parameters in the model can be found in reference (27, 28).

We set the number of subgroups to three during the statistical analysis. To ensure the interpretability and representability of all subgroups, we set a threshold to the minimal prior probability to all subgroups. Specifically, if there exist *m* subgroups and the prior probabilities of these groups are smaller than the given thresholding value, which is 0.2 in this study, all these *m* subgroups will be automatically merged into other subgroups, and the group number will reduce *m*.

### Projection

From the model fitting results of the Poisson mixture regression model, on the one hand, we could obtain the average value of fitted DMFT within different subgroups in 2005 and 2015. On the other hand, based on the data-driven classification results, we could calculate the proportions of different subgroups within each category of risk factor and proportions of different categories for a given risk factor within different subgroups. **Table 2** summarizes the corresponding proportion results. In fact, combining the results in 2005 and 2015, we could derive the projections for the quantities we care about in 2025. Thus, obtaining a two-decades-trend curve, which can help us observe the influence of risk factors on dental caries clearly and intuitively. We will first discuss how the projections in 2025 are calculated. Let *p*_*t, k*_ be the quantity we care about, which can be the mean value of DMFT from subgroup *k* in year *t*, proportion of the *k*th category for a given risk factor in year *t*, or proportion of the *k*th subgroup in year *t* within a given risk factor category. To *p*_*t, k*_, we assume that


$$\begin{array}{l} \log\left(p_{t,k}+1\right)=a_{k}+\log\left(t\right)b_{k}\;,\;\left(2\right) \end{array}$$

for *t* = 2005, 2015, 2025, *k* = 1, …, *K*, and *K* is the total number of subgroups (or number of categories for a risk factor). When *p*_*t, k*_ represents a proportion, as $\overset{K}{\underset{k=1}{\sum }}p_{t,k}=1$, the number of equations in (2) should be *K*−1. Using equations with *t* = 2005 and 2015, we obtain that $a_{k}=\log\left(p_{2005,k}+1\right)-\log\left(2005\right)\frac{\log\left(p_{2015,k}+1\right)-\log\left(p_{2005,k}+1\right)}{\log\left(2015\right)-\log\left(2025\right)}$ and $b_{k}=\frac{\log\left(p_{2015,k}+1\right)-\log\left(p_{2005,k}+1\right)}{\log\left(2015\right)-\log\left(2025\right)}$ for *k* = 1, …, *K*. Then, we have *p*_2025, *k*_ = exp{*a*_*k*_ + log(2025)*b*_*k*_}−1 for *k* = 1, …, *K*. Under the proportion case, we only need to compute proportions *p*_2025, 1_ to *p*_2025, *K*−1_ with the above equations; and the last term can be computed by $p_{2025,K}=1-\overset{K-1}{\underset{k=1}{\sum }}p_{2025,k}$. Combining all proportions *p*_*t, k*_, for *t* = 2005, 2015, 2025 and *k* = 1, …, *K* together, the two-decades-trend curves can be plotted directly.

## Results

Removing all individuals with missing observations, the sample left is 33,402, among which 10,241 are from surveys in 2005, while the other 23,161 are from surveys in 2015.

To determine a suitable number of subgroups, we chose the preselected group number set as *S* = {2, 3, 4} and fitted the Poisson mixture regression model by setting the initial group number as each element of *S*. From the results of the group number selection in Table 1, we found that when the initial group number was larger than 3, according to the prior probability restriction we mentioned above, some subgroups were removed, and only three groups were finally left. Thus, we chose a group number of three.

**Table 1:** Results of group number selection.

**Iteration**	**Convergence**	**Preselected**	**Computed**	**BIC**
**times**		**GN**	**GN**	
44	TRUE	2	2	7427586
84	TRUE	3	3	7424931
80	TRUE	4	3	7423660

In this table, “GN” represents the group number, “Preselected GN” refers to the initial group number we set before model fitting, while the “Computed GN” is the final number of subgroups we obtained from the model fitting results.

The mean values of observed DMFT (SD) for subgroups 1, 2, and 3 were 0.18 (0.56), 1.31 (1.49), and 2.91 (1.89), respectively. It is obvious that the severity of caries risk for these three groups is increasing, which corresponds to the low-risk, moderate-risk, and high-risk groups. In 2005, the population proportions of these three subgroups were 65.2, 25.5, and 9.3%, while the proportions were 64.1, 24.7, and 11.2% in 2015. Compared with 2005, the proportions of subgroups 1 and 2 are smaller, while the proportion in subgroup 3 has an increase. Overall, the oral health status in 2015 was worse than that in 2005.

Except for the above summary information, more detailed information for the results of the Poisson mixture regression model is summarized in Appendix Table A1, which contains the point estimators as well as the confidence intervals for all subgroup-specified effects of all risk factors involved in the model. The results indicate that the relative effects for most risk factors are significant.

To investigate the relationships between subgroups and all risk factors, we calculated two types of proportions. One type of proportion is the proportions of all categories for a given risk factor within different subgroups, which can help us see the composition of each subgroup clearly. The other type of proportion is the proportion of subgroups in each category of a given risk factor. This proportion can reflect the similarity and difference in DMFT status among categories of the risk factor that is of interest. Detailed results of proportions are available in Table 2.

**Table 2:** Population proportions for risk factor-based subgroups.

**Risk factor**	**Categories**	**Subgroup 1 (%)**		**Subgroup 2 (%)**		**Subgroup 3 (%)**		**Total**	
		**2005**	**2015**	**2005**	**2015**	**2005**	**2015**	**2005**	**2015**
Full data		65.2	64.1	25.5	24.7	9.3	11.2	100.0	100.0
Region	East North	6.4 (57.2)	5.2 (53.6)	6.8 (23.8)	7.1 (28.1)	14.8 (19.0)	10.1 (18.3)	7.2 (100.0)	6.2 (100.0)
	North	10.1 (57.2)	10.2 (62.0)	19.4 (42.8)	12.9 (30.5)	0.0 (0.0)	7.0 (7.5)	11.5 (100.0)	10.5 (100.0)
	East	24.4 (56.5)	29.9 (74.5)	48.0 (43.5)	26.6 (25.5)	0.0 (0.0)	0.1 (0.1)	28.2 (100.0)	25.7 (100.0)
	Middle South	37.4 (79.5)	28.0 (59.2)	0.3 (0.3)	27.3 (22.3)	66.7 (20.2)	50.2 (18.5)	30.7 (100.0)	30.3 (100.0)
	West South	12.3 (55.2)	17.3 (58.6)	25.5 (44.8)	17.4 (22.6)	0.0 (0.0)	31.8 (18.8)	14.5 (100.0)	19.0 (100.0)
	West North	9.4 (78.2)	9.4 (73.1)	0.0 (0.0)	8.7 (25.9)	18.5 (21.8)	0.8 (1.0)	7.9 (100.0)	8.3 (100.0)
Census type	Rural	73.6 (67.1)	54.4 (62.1)	64.5 (23.0)	60.0 (26.3)	76.5 (9.9)	58.1 (11.6)	71.5 (100.0)	56.2 (100.0)
	Urban	26.4 (60.6)	45.6 (66.7)	35.5 (31.8)	40.0 (22.5)	23.5 (7.6)	41.9 (10.8)	28.5 (100.0)	43.8 (100.0)
Gender	Female	46.1 (62.8)	42.3 (58.7)	50.8 (27.1)	54.9 (29.3)	52.3 (10.1)	49.7 (12.0)	47.9 (100.0)	46.2 (100.0)
	Male	53.9 (67.4)	57.7 (68.8)	49.2 (24.1)	45.1 (20.7)	47.7 (8.5)	50.3 (10.5)	52.1 (100.0)	53.8 (100.0)
Only child	No	63.7 (68.3)	67.7 (63.3)	50.1 (21.0)	69.2 (25.0)	70.2 (10.7)	71.7 (11.7)	60.8 (100.0)	68.5 (100.0)
	Yes	36.3 (60.4)	32.3 (65.8)	49.9 (32.5)	30.8 (24.1)	29.8 (7.1)	28.3 (10.1)	39.2 (100.0)	31.5 (100.0)
Parents' educational	Never been Educated	1.0 (73.2)	0.5 (60.3)	0.6 (18.0)	0.5 (25.2)	0.8 (8.8)	0.7 (14.5)	0.8 (100.0)	0.5 (100.0)
level	Elementary school	17.0 (68.6)	10.8 (65.5)	12.6 (19.9)	10 (23.1)	20.0 (11.5)	10.8 (11.4)	16.2 (100.0)	10.6 (100.0)
	Middle school	48.5 (66.5)	47.6 (63.7)	46.2 (24.7)	48.6 (25.0)	45.0 (8.8)	48.5 (11.3)	47.6 (100.0)	48.0 (100.0)
	High school	22.0 (64.3)	21.4 (64.1)	23.1 (26.4)	21.2 (24.5)	22.3 (9.3)	21.7 (11.4)	22.3 (100.0)	21.4 (100.0)
	Secondary school	4.3 (58.5)	5.2 (59.7)	5.7 (30.2)	5.9 (26.3)	5.8 (11.3)	6.9 (14.0)	4.8 (100.0)	5.5 (100.0)
	College	4.0 (56.9)	6.7 (64.8)	6.3 (34.7)	6.6 (24.7)	4.2 (8.4)	6.2 (10.5)	4.6 (100.0)	6.6 (100.0)
	Undergraduate	2.8 (58.3)	6.7 (67.4)	4.5 (36.1)	6.3 (24.2)	1.9 (5.6)	4.8 (8.4)	3.2 (100.0)	6.4 (100.0)
	Graduate or higher	0.4 (47.9)	1.1 (71.2)	1.0 (52.1)	0.9 (23.6)	0.0 (0.0)	0.4 (5.2)	0.5 (100.0)	1.0 (100.0)
Tooth brushing	Seldom or never	23.9 (69.6)	12.6 (67.1)	18.1 (20.7)	10.5 (21.7)	23.4 (9.7)	12.0 (11.2)	22.4 (100.0)	12.0 (100.0)
	Daily or often	76.1 (64.0)	87.4 (63.7)	81.9 (21.7)	89.5 (25.1)	76.6 (14.3)	88.0 (11.2)	77.6 (100.0)	88.0 (100.0)
Dentist visit	No	64.7 (77.5)	53.3 (70.3)	26.0 (12.2)	37.1 (18.8)	60.6 (10.3)	47.3 (10.9)	54.5 (100.0)	48.7 (100.0)
	Yes	35.3 (50.5)	46.7 (58.3)	74.0 (41.4)	62.9 (30.2)	39.4 (8.1)	52.7 (11.5)	45.5 (100.0)	51.3 (100.0)

Values in the above table represent the proportions for different categories of a given risk factor within different subgroups, while values in parentheses represent the proportions of subgroups for each category of a given risk factor.

**Figure 1:**
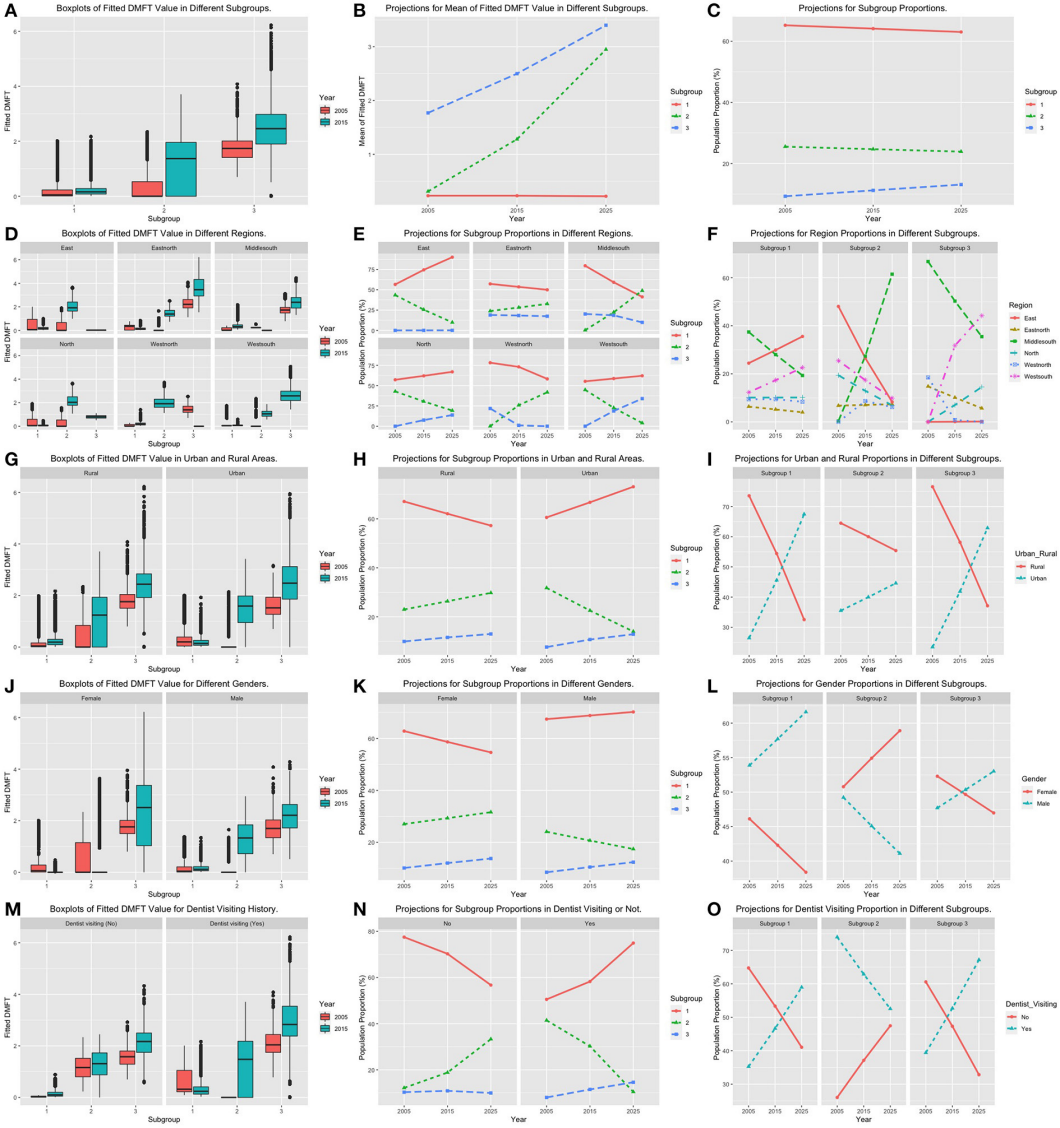
Boxplots for fitted DMFT, projection plots for subgroup proportions and risk factor category proportions. The first column **(A,D,G,J,M)** summarizes the boxplots of fitted DMFT values within subgroups by adjusting different risk factors. The second column **(B,E,H,K,N)** corresponds to the plots of subgroup projections within each risk factor category. The third column **(C,F,I,L,O)** are plots of risk factor category projection within different subgroups.

In Table 2, we also calculated the proportions of these three subgroups within the full population. Even though all these proportions changed significantly from 2005 to 2015, the proportions of the low- and moderate-risk subgroups decreased, and the proportion for the severe-risk group increased. We selected several factors whose proportions changed significantly from 2005 to 2015 for further projection analysis. The region, census type, gender and dentist visiting history were incorporated. To intuitively see the changing trend of the DMFT, we also performed projection analysis on the mean value of the fitted DMFT from the model. All of the above results are summarized in Figure 1.

### Regions

Based on the geographical distribution, we clustered all 31 provinces into six regions: the west north, the east north, the north, the east, the middle south and the west south. Figures 1E,F plot all estimated and projected proportions by adjusting categories of region and subgroup. It is obvious that the trends of caries risk status for different regions are various. The eastern region has the best oral health situation among all regions. The proportion of subgroup 1 was increasing, the proportion of subgroup 2 was decreasing, and the proportion of subgroup 3 was robustly around zero. The patterns for subgroup proportions in the northern region are very similar to those in the eastern region. However, the increasing speed for the population in subgroup 1 in the northern region was much slower than that in the eastern region, and the proportion of subgroup 3 in the north increased with a fast speed from 2005 to 2015. Having a look at Figure 1F, we found that the proportion of the northern region in subgroup 3 continued to increase and will become the third largest one in 2025. The subgroup proportion trends for the east north were the most robust one among all six regions. The proportion of subgroup 3 remained at a relatively high level with a slight decrease. The proportion of subgroup 1 decreased, while the proportion of subgroup 2 increased with a similar speed. The projection values of subgroups 2 and 3 become increasingly close to each other. The patterns of the subgroup trend curves in the middle south and the west north regions are quite similar. Both the proportions in subgroups 1 and 3 decreased significantly, while the proportions of subgroup 2 increased substantially. The projections indicate that the overall situation of the west-north region is better. On the one hand, the projection for the subgroup 3 proportion in the northwestern region has decreased to nearly zero since 2015. On the other hand, the proportion of subgroup 2 in the middle south region will exceed that of subgroup 1 in 2025. Figure 1F also indicates that the middle south region will become the major component of subgroup 2 in 2025. In the west-north region, the proportion of subgroup 1 showed a slight increase. The proportion of subgroup 2 decreased substantially, while the proportion of subgroup 3 increased to a large degree. From the projection results in Figure 1F, we found that the dental caries issue in the southwestern region will become even more severe because it will be the major component of subgroup 3 in 2025.

#### Census type

Figures 1H,I summarize the proportion projection results for populations from different subgroups and different census types. Figure 1H shows that the proportion of subgroup 3 in both rural and urban areas increased with similar speed. The overall situation for the urban region is better because the proportion of subgroup 1 continued to increase, and the projection value in 2025 exceeded 70%. At the same time, the proportion of subgroup 2 in urban areas continued to decrease, and the projection values of subgroups 2 and 3 were only slightly more than 10% in 2025. However, the proportion of subgroup 2 increased rapidly in rural regions. Figure 1I indicates that the rural region will be the major part of subgroup 2 in 2025, while in subgroups 1 and 3, the urban region will become the major component in 2025.

#### Gender

Figures 1K,L correspond to the projection results for male and female populations. From Figure 1K, we know that the trend of the subgroup 3 proportion for both male and female teenagers is similar. Even though the proportion of subgroup 3 in the male population was lower in 2005 and 2015, its projection value in 2025 was very close to that of females. Figure 1L shows that males will become the major component in subgroup 3 in 2025. As the proportion of subgroup 2 decreased and the proportion of subgroup 1 increased, the overall situation of male teenagers was better.

#### Dentist visiting history

The projection results for teenagers with or without a dentist visit history are summarized in Figures 1N,O. On the one hand, from Figure 1N, we realize that the proportion of subgroup 1 had an obvious increase in the group with a dentist visiting history, while it decreased substantially in the group without a dentist visiting history. Compared with the group without dentist visiting experience, the proportion of subgroup 3 in the group with dentist visiting experience increased. However, its overall dental caries status was better because the moderate-risk subgroup decreased with a high speed. Figure 1O also indicates that even the group with dentist visiting experience will be the major part of subgroup 3 in 2025. The advantages of its proportion in subgroups 1 and 2 were still obvious.

In addition to the above four risk factors, we also performed projection analysis on the mean value of the fitted DMFT as well as the proportion of subgroups within the full population. All results are shown in Figures 1B,C. Figure 1B indicates that both the fitted values of DMFT in subgroups 1 and 2 were increased significantly. The projected DMFT values in 2025 of these two groups were very close. Figure 1C shows that the increasing rate of subgroup 3 was the fastest among all subgroups. The proportion of subgroup 2 was relatively stable, while the proportion of subgroup 1 was decreasing. In summary, the situation of dental caries in 2015 was worse than that in 2005, and the situation will become even worse in 2025.

## Discussion

**Figure 2:**
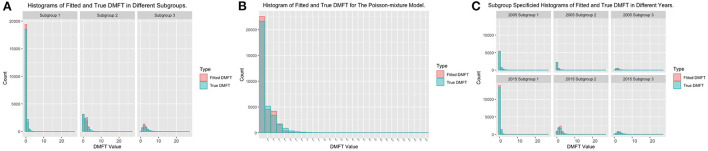
Histograms of model fitting results of the Poisson mixture regression model. **(A)** Histograms of fitted and observed DMFT in different regions. **(B)** Histograms of fitted and observed DMFT for the Poisson mixture regression model. **(C)** Subgroup specified histograms of fitted and observed DMFT in different years.

We compared the model fitting results of the Poisson mixture regression model with those of the zero-inflated Poisson (ZIP) regression model (29–32). To quantify the similarity level between the fitted and observed DMFT values, we calculated the frequency number of the corresponding DMFT taking different integers, which can be found in Table 3. To make the model fitting results more visible, we also plotted the histograms for the observed and the fitted DMFTs from different methods, which are listed in Figure 2. Similar to our previous study (12), all the results illustrate that the Poisson mixture regression model has better goodness-of-fit results than the ZIP model. The number of fitted zeros for the ZIP model is much smaller than the observed ones, while a large proportion of the observed zeros were estimated as one mistakenly by the ZIP model. In addition, the range of the DMFT fitted by the ZIP model is from 0 to 2, which is totally inconsistent with the observed DMFT values. We also tested the model fitting results for the Poisson mixture regression model within different subgroups at different time points, which corresponds to Figure 2C. The fitted distribution of DMFT is very close to the observed distribution in almost all subgroups, which indicates that the Poisson mixture regression model can fit the present dataset better. Thus, the subgroup detection method is a good way to solve the heterogeneity issue existing within the full population. We also calculated the *R*-squared value, which is the ratio of the variance for the predicted and observed DMFT obtained by different models. The *R*-squared results for the Poisson mixture regression model are 0.52, while the results for the ZIP model are only ~0.13. This also testify the advantages of the Poisson mixture regression model.

**Table 3:** Count for observed and fitted values of DMFT by the Poi-mixture and the ZIP model.

**Value**	**0**	**1**	**2**	**3**	**4**	**≥5**
Observed DMFT	21,660	5,152	3,392	1,565	849	784
ZIP model	8,425	23,714	1,263	0	0	0
Poi-mixture model	22,572	4,514	4,161	1,700	354	101

Each value in this table represents the count of the corresponding variable taking the integer value.

**Figure 3:**
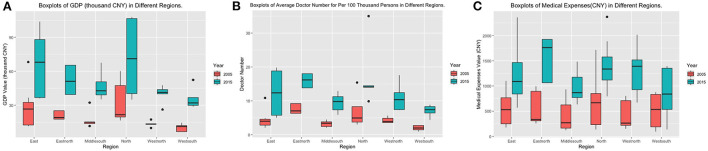
Boxplots for medical care and economic status within different regions. **(A)** Boxplot of GDP within different regions in China. **(B)** Boxplot of the average number of doctors for per 100,000 persons within different regions in China. **(C)** Boxplot for medical expenses within different regions in China.

Returning to the projection analysis results for the risk factor region, the results in Figures 1E,F indicate that the trends for caries risk in different regions vary. One reason is that the lifestyles for different regions exhibit significant differences. Another reason may be highly correlated with economic ability and medical policy within different regions. We used the GDP value, the average doctor numbers, and the average medical expenses in different regions to measure the region-specified economics and the medical care abilities (11). The summary information and boxplots for these factors are summarized in Table 4 and Figure 3. Among all six regions, the northern and eastern regions have the highest GDP and similar medical ability. The medical expense in the eastern region is lower than that in the northern region, and the oral health status for the eastern region is also better than that of the northern region. The difference between the projection trends for these two regions mainly focuses on high-risk subgroup 3. The proportion of subgroup 3 in the eastern region was approximately zero, while that in the northern region increased to almost 10% in 2015. The trends for the low-risk and moderate-risk subgroups for the eastern and northern regions are similar. Except for the north and east regions, the GDP in the east-north region is the highest among all four regions. At the same time, the average number of doctors and medical expenses in the east-north region are the highest among all six regions, which indicates that this region paid much attention to the medical care problem (13, 33). Even though the proportion of subgroup 3 in the east north is robustly around a relatively high level, the trends for the other two subgroups did not have significant change, and the overall situation of dental caries was controlled well. Both the GDP level and the average number of doctors in the western north and the middle south regions are similar. In 2015, the medical expenses in the northwest region significantly increased. At the same time, the proportion of subgroup 3 in the northwest also decreased greatly, which indicates that higher attention to medical care is efficient in controlling dental caries status. The west south region has the lowest GDP as well as medical care ability among all six regions. The proportion of subgroup 3 in the southwestern region also increased greatly during the past decades. The status of oral health in this region is the worst in China (34, 35). In addition to the economic and medical care ability, the poor oral health behavior in this region may be another reason. In summary, balancing the economic and medical care development among different regions is an important element in improving the oral health status in China. For the lifestyle difference issue between regions, advertising the importance of oral healthy as well as healthy lifestyle to adolescents is a good idea.

**Table 4:** Summary information for medical care and economic status in different regions.

**Region**	**GDP (thousand CNY)**		**Doctor number**		**Medical expense (CNY)**	
	**2005**	**2015**	**2005**	**2015**	**2005**	**2015**
East	29.06 (16.50)	67.76 (23.56)	4.43 (2.49)	12.18 (5.25)	524 (292)	1,207 (440)
East North	20.56 (3.19)	52.07 (10.40)	7.50 (1.23)	16.10 (1.71)	593 (304)	1,477 (398)
Middle South	16.65 (6.50)	46.74 (11.36)	3.28 (0.80)	9.42 (2.43)	420 (265)	1,001 (263)
North	33.11 (17.75)	73.20 (31.40)	7.16 (4.67)	17.88 (9.16)	673 (516)	1,408 (452)
West North	13.24 (2.37)	39.43 (7.31)	4.21 (0.79)	11.09 (3.87)	453 (253)	1,262 (420)
West South	10.84 (2.88)	36.22 (8.87)	1.94 (0.76)	7.10 (1.58)	438 (295)	867 (420)

The unit of GDP in this table is one thousand CNY, and the doctor number refers to the average doctor number per hundred thousand persons within each region, and values in parentheses represent the corresponding standard deviation.

The census type has a significant influence on caries risk (36). The oral health status in the urban group has obvious advantages over that in the rural group. The prevalence of dental caries in rural areas is slowly increasing, while the trend of polarization is showing in urban areas. Thus, minimizing the difference between urban-rural areas and assessing caries risk to find high-risk groups in urban areas are the key elements for improving the oral health situation for teenagers. The risk patterns between the male and female groups were also different. In 2005, advantages for the male group were obvious. This is similar to the results of several cohort and cross-sectional studies showing that girls have a higher dental caries level than boys (18, 37, 38). In 2015, the proportion of males in the high-risk subgroup increased considerably, which was almost the same as the proportion of females in 2015. These results suggest that the inequalities between men and women were decreasing in Chinese teenagers. The results for risk factor dentist visiting history are very interesting. In 2005, the oral health status for the group without dentist visiting experience was obviously better than that of the other group. From the projection results, the proportion of dentists visiting in low- and high-caries groups will increase substantially in 2025. The results indicated that the utilization of dental services increased both in low- and high-caries risk groups. One possible explanation is that the Chinese government implemented the National Oral Health Comprehensive Intervention Program for children since 2008 to reduce dental caries. Our previous study showed a significant difference in the dental caries status and oral health behaviors of the 12-year-old children in the program-covered and uncovered regions (8). Thus, it is also very important for teenagers to visit the dentist and accept treatments in a timely manner (39).

In this paper, we analyzed the heterogeneous associations between risk factors and the DMFT score in the cross-sectional dataset by fitting the Poisson mixture regression model, which can detect subgroups of population and fits the subgroup-specified models simultaneously. We tested the statistical significance on the heterogenous associations and performed projection analysis on the future DMFT, which provides insights into the primary reasons for dental caries as well as the factor-based development trend of DMFT. Thus, our analysis results lead to some constructive solutions for improving dental health status. We conclude that region difference is the principal factor for DMFT's diversified changes. On the one hand, it is essential to balance the economic and medical abilities among different regions. On the other hand, it is also important to strengthen the universal of oral health knowledge among adolescents. However, this study also has some limitations. All conclusions in this paper were obtained from the survey data at two time points. Using data collected at multiple time points can help us identify the time varying changes in dental caries with higher accuracy. We leave this problem to further research.

## Data availability statement

The raw data supporting the conclusions of this article will be made available by the authors, without undue reservation.

## Ethics statement

Ethical clearance was approved by the Stomatological Ethics Committee of the Chinese Stomatological Association. Written informed consent to participate in this study was provided by the participants' legal guardian/next of kin.

## Author contributions

JH, HL, JK, and CY contributed to conception, design, data acquisition, analysis, interpretation, drafted, and critically revised the manuscript. All authors gave final approval and agree to be accountable for all aspects of the work.

## Funding

This study was funded by Peking University School and Hospital of Stomatology (grant number: PKUSSNCT-20Y02), Peking University International Strategic Partnership Fund, and Scientific Research Fund of National Health Commission of the People's Republic of China (201502002).

## Conflict of interest

The authors declare that the research was conducted in the absence of any commercial or financial relationships that could be construed as a potential conflict of interest.

## Publisher's note

All claims expressed in this article are solely those of the authors and do not necessarily represent those of their affiliated organizations, or those of the publisher, the editors and the reviewers. Any product that may be evaluated in this article, or claim that may be made by its manufacturer, is not guaranteed or endorsed by the publisher.
