# Supplementary material for: Subgroup detection-based dental caries status and inequalities trend exploration: A nationwide, 10-year-repeated cross-sectional study
Source: Front Public Health. 2022 Aug 12;10:916878. doi: 10.3389/fpubh.2022.916878 (PMC9412197; doi:10.3389/fpubh.2022.916878)
Supplement: Supplementary file 1 [file Data_Sheet_1.docx]

**Appendix**

The following is a brief introduction to the model and some notations used in this manuscript. Denote all DMFT scores collected in 2005 and 2015 as the response $y$, which ranges in count values from 0 to 28. $\mathbf{x}$ represents a vector composed of all risk factors we are interested in. The Poisson mixture regression model assumes that the conditional distribution of $y$ given $\mathbf{x}$ follows a mixture Poisson distribution with the expression

$$H\left( y | \mathbf{x},\boldsymbol{\omega}; \right)=\sum_{k=1}^{K} \pi_{k} \mathrm{Poi}\left\{ {y|\lambda}_{k}\left( \mathbf{x} \right) \right\},$$

where $\pi_{k}$is the probability weight of the response classified into subgroup $k$, and $\mathrm{Poi}\left\{ {y|\lambda}_{k}\left( \mathbf{x} \right) \right\}$ is the Poisson probability mass function with mean intensity $\lambda_{k}\left( \mathbf{x} \right)$. Specifically, we model the mean intensity $\lambda_{k}\left( \mathbf{x} \right)$ by the Poisson regression model

$\log\left\{ \lambda_{k}\left( \mathbf{x}\boldsymbol{,}\boldsymbol{\beta}^{\left( k \right)} \right) \right\}=\sum_{j=1}^{p} \beta_{j}^{\left( k \right)}x_{j}, k=1,\ldots,K,$ (1)

where $\beta_{j}^{\left( k \right)}$ denotes the effect of $x_{j}$ in subgroup $k$; for $j=1,\ldots,p$ and $k=1,\ldots,K$, $K$ is the total number of subgroups, and $x_{j}$, the $j$th entry of $\mathbf{x}$, refers to the $j$th risk factor.

**Appendix Table 1**: Relative effect estimations and inference for all risk factors.

|  |  | **Subgroup1** | **Subgroup2** | **Subgroup3** |
| --- | --- | --- | --- | --- |
| **Region** | West North |  |  |  |
|  | East North | **2005:** 1.010 (0.925, 1.096) | **2005:** 1.450 (1.329, 1.572) | **2005:** 0.527 (0.506, 0.548) |
|  |  | **2015:** -0.430 (-0.479, -0.381) | **2015:** -0.385 (-0.404, -0.365) | **2015:** 7.529 (6.604, 8.453) |
|  | North | **2005:** 1.939 (1.852, 2.027) | **2005:** 1.718 (1.604, 1.832) | **2005:** -5.092 (-5.901, -4.284) |
|  |  | **2015:** -1.129 (-1.198, -1.060) | **2015:** 0.025 (0.015, 0.035) | **2015:** 5.796 (4.870, 6.722) |
|  | East | **2005:** 1.993 (1.961, 2.080) | **2005:** 1.969 (1.756, 2.083) | **2005:** -5.276 (-5.888, -4.663) |
|  |  | **2015:** -0.182 (-0.211, -0.152) | **2015:** -0.018 (-0.026, -0.009) | **2015:** 2.065 (1.113, 3.018) |
|  | Middle South | **2005:** 0.384 (0.301, 0.467) | **2005:** 0.364 (0.256, 0.472) | **2005:** 0.184 (0.167, 0.200) |
|  |  | **2015:** 0.759 (0.715, 0.803) | **2015:** -15.689 (-42.187, 10.808) | **2015:** 7.157 (6.232. 8.081) |
|  | West South | **2005:** 1.843 (1.756, 1.931) | **2005:** 2.291 (2.177, 2.406) | **2005:** -15.851 (-66.234, 34.531) |
|  |  | **2015:** -0.942 (-0.987, -0.897) | **2015:** -0.672 (-0.690, -0.653) | **2015:** 7.264 (6.340, 8.189) |
| **Census Type** | Rural-0, Urban-1 | **2005:** -0.009 (-0.024, 0.004) | **2005:** 0.029 (0.007, 0.052) | **2005:** -0.255 (-0.270, -0.240) |
|  |  | **2015:** -0.431 (-0.354, -0.328) | **2015:** -0.088(-0.094, -0.081) | **2015:** 0.012 (0.006, 0.018) |
| **Gender** | Female-0, Male-1 | **2005:** -0.377 (-0.390, -0.365) | **2005:** -0.649 (-0.665, -0.633) | **2005:** -0.063 (-0.075, -0.051) |
|  |  | **2015:** -0.757 (-0.771, -0.743) | **2015:** -0.330 (-0.337, -0.324) | **2015:** -0.322 (-0.328, -0.316) |
| **Only Child** | No-0, Yes-1 | **2005:** 0.081 (0.068, 0.094) | **2005:** -0.034 (-0.051, -0.017) | **2005:** -0.266 (-0.280, -0.251) |
|  |  | **2015:** -0.434 (-0.452, -0.416) | **2015:** -0.055 (-0.061, -0.048) | **2015:** -0.111 (-0.118, -0.104) |
| **Parents Education Level** | 1-8, from low to high | **2005:** 0.0145 (0.010, 0.019) | **2005:** -0.053 (-0.062, -0.045) | **2005:** 0.071 (0.065, 0.076) |
|  |  | **2015:** -0.067 (-0.072, 0.062) | **2015:** -0.003 (-0.005, -0.001) | **2015:** -0.019 (-0.021, -0.016) |
| **Tooth Bushing** | Not daily 0**,** daily 1 | **2005:** 0.155 (0.137, 0.173) | **2005:** -0.150 (-0.168, -0.133) | **2005:** 0.191 (0.176, 0.205) |
|  |  | **2015:** 0.273 (0.250, 0.296) | **2015:** -0.008 (-0.092, -0.073) | **2015:** 0.072 (0.062, 0.082) |
| **Dentist Visit History** | No-0, Yes-1 | **2005:** 3.150 (3.072, 3.228) | **2005:** -10.619 (-15.173, -6.066) | **2005:** 0.339 (0.326, 0.351) |
|  |  | **2015:** 0.970 (0.954, 0.986) | **2015:** 0.426 (0.420, 0.433) | **2015:** 0.398 (0.392, 0.405) |
| **Knowledge Score** | 0-8, from bad to good | **2005:** 0.051 (0.049, 0.054) | **2005:** 0.022 (0.019, 0.026) | **2005:** -0.034 (-0.037, -0.031) |
|  |  | **2015:** -0.006 (-0.010, -0.003) | **2015:** -0.001 (-0.003, 0.001) | **2015:** 0.009 (0.007, 0.011) |
| **Sugar Intake Score** | 1-16, from low to high | **2005:** -0.005 (-0.007, -0.004) | **2005:** -0.010 (-0.012, -0.008) | **2005:** 0.008 (0.006, 0.009) |
|  |  | **2015:** 0.038 (0.036, 0.040) | **2015:** 0.023 (0.022, 0.024) | **2015:** 0.024 (0.023, 0.025) |
| **Pit-and-fissure Sealant** | No-0, Yes-1 | **2005:** -0.133 (-0.173, -0.093) | **2005:** 1.290 (0.818, 1.762) | **2005:** -0.065 (-0.142, 0.011) |
|  |  | **2015:** 0.548 (0.523, 0.572) | **2015:** -0.138 (-0.153, -0.123) | **2015:** -0.242 (-0.257, -0.226) |

**Note**: Estimated relative effects and corresponding confidence intervals for all risk factors in the Poisson mixture regression model.

**Appendix Table 2:** p value of hypothesis relative effects of each risk factor in 2005 and 2015.

| **Risk Factor** | **Subgroup1** | **Subgroup2** | **Subgroup3** |
| --- | --- | --- | --- |
| **Region** (Baseline: West North) |  |  |  |
| East North | <2.2e-16 | <2.2e-16 | <2.2e-16 |
| North | <2.2e-16 | <2.2e-16 | <2.2e-16 |
| East | <2.2e-16 | <2.2e-16 | <2.2e-16 |
| Middle South | <2.2e-16 | <2.2e-16 | <2.2e-16 |
| West South | <2.2e-16 | <2.2e-16 | 0.369 |
| **Census Type** | <2.2e-16 | <2.2e-16 | <2.2e-16 |
| **Gender** | <2.2e-16 | <2.2e-16 | <2.2e-16 |
| **Only Child** | <2.2e-16 | 0.016 | <2.2e-16 |
| **Parents Education Level** | <2.2e-16 | <2.2e-16 | <2.2e-16 |
| **Tooth Bushing** | <2.2e-16 | 1.6e-14 | <2.2e-16 |
| **Dentist Visit History** | <2.2e-16 | 2.0e-06 | <2.2e-16 |
| **Knowledge Score** | <2.2e-16 | <2.2e-16 | <2.2e-16 |
| **Sugar Intake Score** | <2.2e-16 | <2.2e-16 | <2.2e-16 |
| **Pit-and-fissure Sealant** | <2.2e-16 | 3.0e-09 | 6.3e-06 |

**Appendix Table 3:** p value of hypothesis for relative effects of each risk factor among all subgroups in 2005 in 2015.

| **Risk Factor** | **2005** | **2015** |
| --- | --- | --- |
| **Region** (Baseline: West North) |  |  |
| East North | <2.2e-16 | <2.2e-16 |
| North | <2.2e-16 | <2.2e-16 |
| East | <2.2e-16 | <2.2e-16 |
| Middle South | 8.4e-08 | <2.2e-16 |
| West South | 6.0e-10 | <2.2e-16 |
| **Census Type** | <2.2e-16 | <2.2e-16 |
| **Gender** | <2.2e-16 | <2.2e-16 |
| **Only Child** | <2.2e-16 | <2.2e-16 |
| **Parents Education Level** | <2.2e-16 | <2.2e-16 |
| **Tooth Bushing** | <2.2e-16 | <2.2e-16 |
| **Dentist Visit History** | <2.2e-16 | <2.2e-16 |
| **Knowledge Score** | <2.2e-16 | <2.2e-16 |
| **Sugar Intake Score** | <2.2e-16 | <2.2e-16 |
| **Pit-and-fissure Sealant** | 4.0e-10 | <2.2e-16 |

**Appendix Table 4**: p values for the population proportion test for risk factor-based subgroups.

| **Risk Factor** | **Categories** | **Subgroup1(%)** | | **Subgroup2(%)** | | **Subgroup3(%)** | |
| --- | --- | --- | --- | --- | --- | --- | --- |
|  |  | **2005** vs **2015** | **2015** vs **2025** | **2005** vs **2015** | **2015** vs **2025** | **2005** vs **2015** | **2015** vs **2025** |
| **Full Data** |  | 1.00 | 1.00 | 1.00 | 1.00 | 0.81 | 0.83 |
| **Region** | East North | 0.78(1.00) | 0.67(1.00) | 0.63(1.00) | 0.54(1.00) | 1.00(0.39) | 1.00(0.43) |
|  | North | 0.56(1.00) | 0.55(1.00) | 0.08(0.34) | 0.10(0.24) | 0.04(0.07) | 0.26(0.11) |
|  | East | 0.02(0.43) | 0.06(0.45) | 0.01(3.49e-3) | 0.01(8.09e-4) | 1.00(1.00) | 1.00(1.00) |
|  | Middle South | 2.13e-3(0.23) | 0.02(0.18) | 9.29e-6(3.49e-7) | 1.22e-4(2.59e-6) | 1.00(0.02) | 0.11(0.05) |
|  | West South | 0.67(0.42) | 0.77(0.38) | 1.72e-3(0.22) | 1.96e-4(0.21) | 6.15e-5(1.10e-8) | 0.03(0.11) |
|  | West North | 0.51(1.00) | 0.04(1.00) | 6.83e-7(0.02) | 0.03(0.59) | 9.29e-6(1.14e-4) | 1.00(1.00) |
| **Census Type** | Rural | 0.56(5.13e-3) | 0.57(4.34e-3) | 0.74(0.66) | 0.64(0.57) | 0.82(6.58e-3) | 1.00(4.63e-3) |
|  | Urban | 0.46(5.13e-3) | 0.44(4.34e-3) | 0.21(0.66) | 0.15(0.57) | 0.63 (6.58e-3) | 0.83(4.63e-3) |
| **Gender** | Female | 0.66(0.67) | 0.67(0.67) | 0.87(0.68) | 0.76(0.67) | 0.82(0.89) | 0.83(0.78) |
|  | Male | 0.88(0.67) | 1.00(0.67) | 0.73(0.68) | 0.59(0.67) | 0.80(0.89) | 0.82(0.78) |
| **Dentist Visit** | No | 0.26(0.11) | 0.08(0.12) | 0.24(0.14) | 0.04(0.20) | 1.00(0.07) | 1.00(0.06) |
|  | Yes | 0.39(0.11) | 0.02(0.12) | 0.14(0.14) | 7.83e-4(0.20) | 0.63(0.07) | 0.53(0.06) |

**Note**: Values in the above table represent the p values for different categories of a given risk factor within different subgroups, while values in parentheses represent the p values of subgroups for each category of a given risk factor.
